# Establishment of a Canine Rabies Burden in Haiti through the Implementation of a Novel Surveillance Program

**DOI:** 10.1371/journal.pntd.0004245

**Published:** 2015-11-24

**Authors:** Ryan M Wallace, Hannah Reses, Richard Franka, Pierre Dilius, Natael Fenelon, Lillian Orciari, Melissa Etheart, Apollon Destine, Kelly Crowdis, Jesse D Blanton, Calvin Francisco, Fleurinord Ludder, Victor Del Rio Vilas, Joseph Haim, Max Millien

**Affiliations:** 1 United States Centers for Disease Control and Prevention, Poxvirus and Rabies Branch, Atlanta, Georgia, United States of America; 2 Ministère de l'Agriculture, des Resources Naturelles et du Développement Rural, Department of Animal Health, Port au Prince, Republic of Haiti; 3 Ministère de la Santé Publique et de la Population, Department of Epidemiology and Laboratory Research, Port au Prince, Republic of Haiti; 4 United States Centers for Disease Control and Prevention, Haiti Office, Port au Prince, Republic of Haiti; 5 Christian Veterinary Mission, Port-au-Prince, Republic of Haiti; 6 Pan American Foot and Mouth Disease Center of the Pan American Health Organization (PAHO), Rio, Brazil; University of Minnesota, UNITED STATES

## Abstract

The Republic of Haiti is one of only several countries in the Western Hemisphere in which canine rabies is still endemic. Estimation methods have predicted that 130 human deaths occur per year, yet existing surveillance mechanisms have detected few of these rabies cases. Likewise, canine rabies surveillance capacity has had only limited capacity, detecting only two rabid dogs per year, on average. In 2013, Haiti initiated a community-based animal rabies surveillance program comprised of two components: active community bite investigation and passive animal rabies investigation. From January 2013 –December 2014, 778 rabies suspect animals were reported for investigation. Rabies was laboratory-confirmed in 70 animals (9%) and an additional 36 cases were identified based on clinical diagnosis (5%), representing an 18-fold increase in reporting of rabid animals compared to the three years before the program was implemented. Dogs were the most frequent rabid animal (90%). Testing and observation ruled out rabies in 61% of animals investigated. A total of 639 bite victims were reported to the program and an additional 364 bite victims who had not sought medical care were identified during the course of investigations. Only 31% of people with likely rabies exposures had initiated rabies post-exposure prophylaxis prior to the investigation. Rabies is a neglected disease in-part due to a lack of surveillance and understanding about the burden. The surveillance methods employed by this program established a much higher burden of canine rabies in Haiti than previously recognized. The active, community-based bite investigations identified numerous additional rabies exposures and bite victims were referred for appropriate medical care, averting potential human rabies deaths. The use of community-based rabies surveillance programs such as HARSP should be considered in canine rabies endemic countries.

## Author Summary

The Republic of Haiti has highest estimated burden of human rabies in the Western Hemisphere, at 130 estimated human deaths annually. Rabies surveillance systems in the majority of the developing world, including Haiti, are ineffective, resulting in underreporting of cases and contributing to the further neglect of this disease. In 2013 a passive rabies surveillance program was implemented in three of Haiti’s 140 communes near the nation’s capital city. Four animal rabies surveillance officers conducted 778 suspect animal rabies investigations in a two-year period and on average found a rabid animal for every 7.4 investigations. Prior to the implementation of this surveillance program Haiti reported an average of two canine and seven human rabies cases each year, for the entire country. This program identified 70 rabid animals and an additional 36 probable rabid animals in only a selected area of the country. These 106 cases represent an 18-fold increase in animal rabies reporting in Haiti. These findings support that canine rabies is a significant burden in Haiti and present data that can be used to improve human rabies burden estimations and enhance canine rabies control efforts.

## Introduction

Rabies is the deadliest of all zoonotic diseases, responsible for more than 59,000 human deaths, annually [1, 2]. It is also the most lethal infectious disease, with a case fatality rate of nearly 100% even with advanced medical intervention [1]. Although all mammals are susceptible to rabies virus infection, only certain reservoir species are capable of maintaining enzootic circulation through conspecific transmission. Reservoir species for the rabies virus include bats in the Western Hemisphere and at least 15 terrestrial mammals worldwide. While all reservoir species can transmit rabies to people, none are more significant than the domesticated dog, which is responsible for nearly all human rabies deaths [2].

In most of the developing world, rabies surveillance methods are ineffective, resulting in underreporting of human and animal cases [3]. The costs of developing and implementing a comprehensive surveillance system for rabies are prohibitive in many developing countries. The methods of rabies surveillance practiced in many countries suffer from fundamental problems including a lack of trained professionals and a lack of diagnostic laboratory capacity capable of providing results clinically relevant for human exposures [3, 4]. In the absence of systems capable of timely investigation and reporting for clinical and public health purposes, there are often few perceived incentives for conducting rabies surveillance. This results in a lack of awareness of case burden, reduced funding for control, and poor community engagement around prevention [3, 4]. This is often referred to as the ‘cycle of neglect’ and contributes to the poor understanding of the global canine rabies burden as well as the continued lack of funding for its control and prevention.

Due to this dearth of surveillance data, estimation methods must be periodically conducted to understand the national and global scope of rabies burden [2]. Multiple burden estimation methods have been attempted, all of which have placed Haiti amongst the highest for canine and human rabies in the Western Hemisphere [2, 5–7]. However, inferences from these global methods on disease burden at fine geographical resolution are limited by poor data availability. Routine passive rabies surveillance systems can provide this region-specific data which integral to inform rabies control activities. Surveillance for rabies provides additional benefits, including the removal of rabid animals from the community by trained professionals, reduction of rabies exposures, and evidence-based use of rabies biologics [8].

Canine rabies has been eliminated in most developed countries, largely due to successful rabies vaccination programs and responsible dog population management [1, 4, 9, 10]. The most recent reduction in the burden of canine rabies has been observed in the Western Hemisphere, where it reached historically low levels as of 2014 [6]. The Republic of Haiti, a Caribbean nation of 10.5 million people, has been identified as one of only several countries in the Western Hemisphere in which these advances in canine rabies control have not been mirrored. The true incidence of human and canine rabies in Haiti is currently not known, however, limited surveillance activity from 2009–2012 detected an average of four canine and seven human rabies cases annually [6, 7].

In 2011, a laboratory-based animal rabies surveillance program was developed to improve our knowledge of the canine rabies burden in Haiti and enact community-level preventive measures. This report details the process of developing the program, data of animal rabies cases and bites reported through this program, and a comparison to the pre-surveillance period of 2009–2012.

## Methods

The Haiti Animal Rabies Surveillance Program (HARSP) was initially conceived in 2011 under the leadership of the Ministère de l'Agriculture, des Ressources Naturelles et du Développement Rural (MARNDR), in collaboration with the Ministère de la Santé Publique et de la Population (MSPP), Christian Veterinary Mission (CVM) and the United States Centers for Disease Control and Prevention (CDC). The HARSP was enacted in three stages from 2011–2013, detailed below. Historical records for animal rabies at the national level were available from September 2009 through December 2012 from MARNDR records.

### Stage One: Laboratory Development

Prior to 2012, all rabid animals in Haiti were diagnosed by Seller’s stain, a method with lower sensitivity and specificity compared to the international standard Direct Fluorescent Antibody test (DFA). During a 24 month period from 2011 through 2012 CDC assisted in the establishment of an animal rabies diagnostic facility at the MARNDR—National Veterinary Laboratory. The laboratory was established in a dedicated room within the virology unit and was outfitted with one fluorescent microscope, an incubator, freezer, fume hood, and supplies required for processing and diagnosing samples. Staff were trained in the method of DFA and Direct Rapid Immunohistochemistry Test (DRIT) tests. Proficiency of testing was conducted through confirmatory testing of samples at CDC and through participation in the Latin American diagnostic proficiency testing program.

### Stage Two: Training of Animal Surveillance Officers

In December of 2012 and June of 2013 trainings were held on principles of rabies education and prevention. Trainings encompassed both classroom and field coursework. Eighteen participants were selected by MARNDR for training and had a background in veterinary education at either the para-professional (nine-week training program) or technician level (two-year degree). In January 2013, one veterinary technician began surveillance activities under HARSP in the Petionville commune, within the West Department. In June 2013, three veterinary para-professionals joined HARSP. The four selected animal rabies surveillance officers (ARSO) received rabies pre-exposure vaccination, training in animal rabies surveillance and bite investigation, and were provided equipment for safe and humane animal capture.

### Stage Three: HARSP Implementation

**Fig 1:**
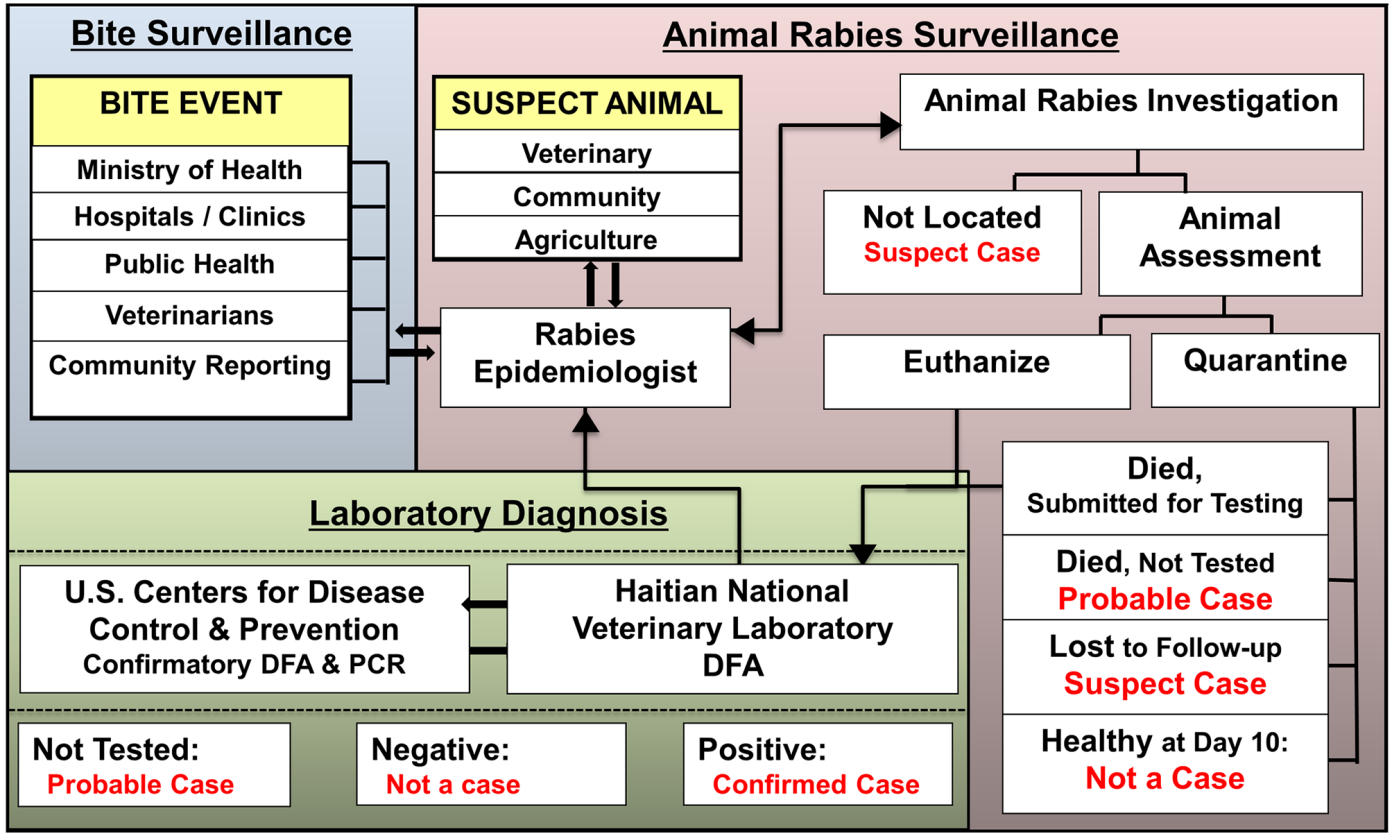
Haiti Animal Rabies Surveillance Program structure for reporting, investigation, and diagnostic testing of rabies suspect animals. *ARSO: Animal Rabies Surveillance Officer.

Rabies is only detectable by current laboratory methods after the virus has infected neurons in the brain stem and cerebellum and it is during this time that the animal will show signs of illness. Therefore, passive surveillance efforts targeting animals which are involved in a bite event or have clinical signs are more likely to detect rabid animals. In Haiti, bites recorded at sentinel hospitals are notifiable events to MSPP–Department of Epidemiology and Laboratory Research; therefore HARSP relies upon inter-ministerial collaborations for animal surveillance in which MSPP reports bites daily to MARNDR for investigation. Summary reports of rabies investigations are shared at weekly One Health meetings. The HARSP also utilizes community, non-sentinel hospital, and veterinary-based reporting of rabies suspect animals for investigation (Fig 1). No formal media campaigns were enacted to advertise HARSP to these stakeholders, rather they were informed through word-of-mouth and engagement during community-based investigations.

Haiti is administratively comprised of ten departments which are divided into 140 communes [11]. Four ARSOs were employed through MARNDR to operate primarily within three communes in the West Department, the most developed of the ten departments; Petionville, Carrefour, and Croix-des-Bouquets. Communes were selected based on proximity to the MARNDR National Veterinary Laboratory and adequate road infrastructure to facilitate travel during investigations. Petionville and Carrefour are communes adjacent to Port-au-Prince. Petionville is considered an affluent commune with a population of 342,694 people [12]. Carrefour is an impoverished commune with a population of 465,019 people [12]. Croix-des-Bouquets is located 13 miles north from the capital city and has a population of 84,812 people [12]. The overall population size targeted by HARSP was c. 1 million (or 8.5% of Haiti’s population). These three communes were the centers for operations for ARSO’s, but investigations were conducted in other communes as time and resources allowed.

HARSP investigations occurred during a two-year surveillance period (January 2013 to December 2014). Investigations were composed of two components: community bite investigation and animal rabies investigation. Animals that appeared healthy were placed on a 14-day in-home observation with both verbal and written instruction on proper animal care and rabies exposure prevention (animal quarantine facilities are not available in Haiti). Animals healthy after 14 days were released from observation. Animals that displayed signs consistent with rabies during the investigation or the observation period were anesthetized and euthanized according to American Veterinary Medical Association standards, utilizing sedation with a ketamine/xylazine combination and euthanasia by intracardiac potassium chloride [13]. Rabies suspect animals that were euthanized or found dead were tested at the MARNDR National Veterinary Laboratory. Investigation results were reported by telephone to the bite victim, the reporting health facility, and the MSPP–Department of Epidemiology and Laboratory Resources. Additional persons identified as exposed during the course of community bite investigations were referred to nearby medical facilities for rabies post-exposure prophylaxis (PEP) evaluation. HARSP investigation forms collected information on the overall health status of the animals, presenting clinical signs, health history, and human exposures. Monitoring of adherence to rabies PEP is not collected in Haiti nor was it collected by HARSP.

### Active Rabies Surveillance

Active surveillance was conducted through convenience sampling of found-dead dogs from June 2013 through December 2014. During this time, US embassy staff, during routine driving activities in the West Department, retrieved found-dead dogs for rabies testing. This type of sampling is assumed to be less biased compared to post-bite animal rabies surveillance, since it does not rely upon symptomatic indicators for investigation [8].

### Surveillance Case Definitions

A case definition was developed to assign a case status to animals investigated through HARSP (Box 1).

**BOX 1: Haiti Animal Rabies Surveillance Program Case Definitions**

- **Confirmed Rabies Cases**
  - Diagnostic confirmation of rabies virus by DFA
- **Probable Rabies Cases**
  - Animals that were not tested for rabies
    AND
  - Died during observation
    OR
  - Did not pass observation (escaped animals, not found by further investigation)
    OR
  - Developed one or more clinical sign and died after being bitten by a suspect/probable/confirmed rabid animal
- **Suspect Rabies Cases**
  - Animals reported to Animal Rabies Surveillance Officers that could not be assessed
    OR
  - Animals that had less than 2 signs of rabies and test results were inconclusive
- **Non-cases**
  - Animals that are healthy after the 14-day observation period
    OR
  - Animals that test negative by DFA

### Data Collection and Analysis

Investigation and diagnostic data collected from January 2013 through December 2014 were compared with historical records of animal rabies cases collected by MARNDR dating back to September 2009. Data were entered into a Microsoft Access database and exported to SAS (version 9.3, SAS Institute Inc., Cary, NC, USA). Univariate, descriptive analysis for temporal and spatial trends were conducted. Odds ratio (OR) and conditional maximum likelihood estimate test of association between clinical signs and case definition (i.e. confirmed case, probable case, suspect case, or non-case) were examined to validate the case definitions and determine factors significantly associated with rabid animals. A formal waiver was obtained from the National Center for Emerging Zoonotic Infectious Diseases human subject’s advisor; this work was deemed exempt, non-research.

## Results

**Fig 2:**
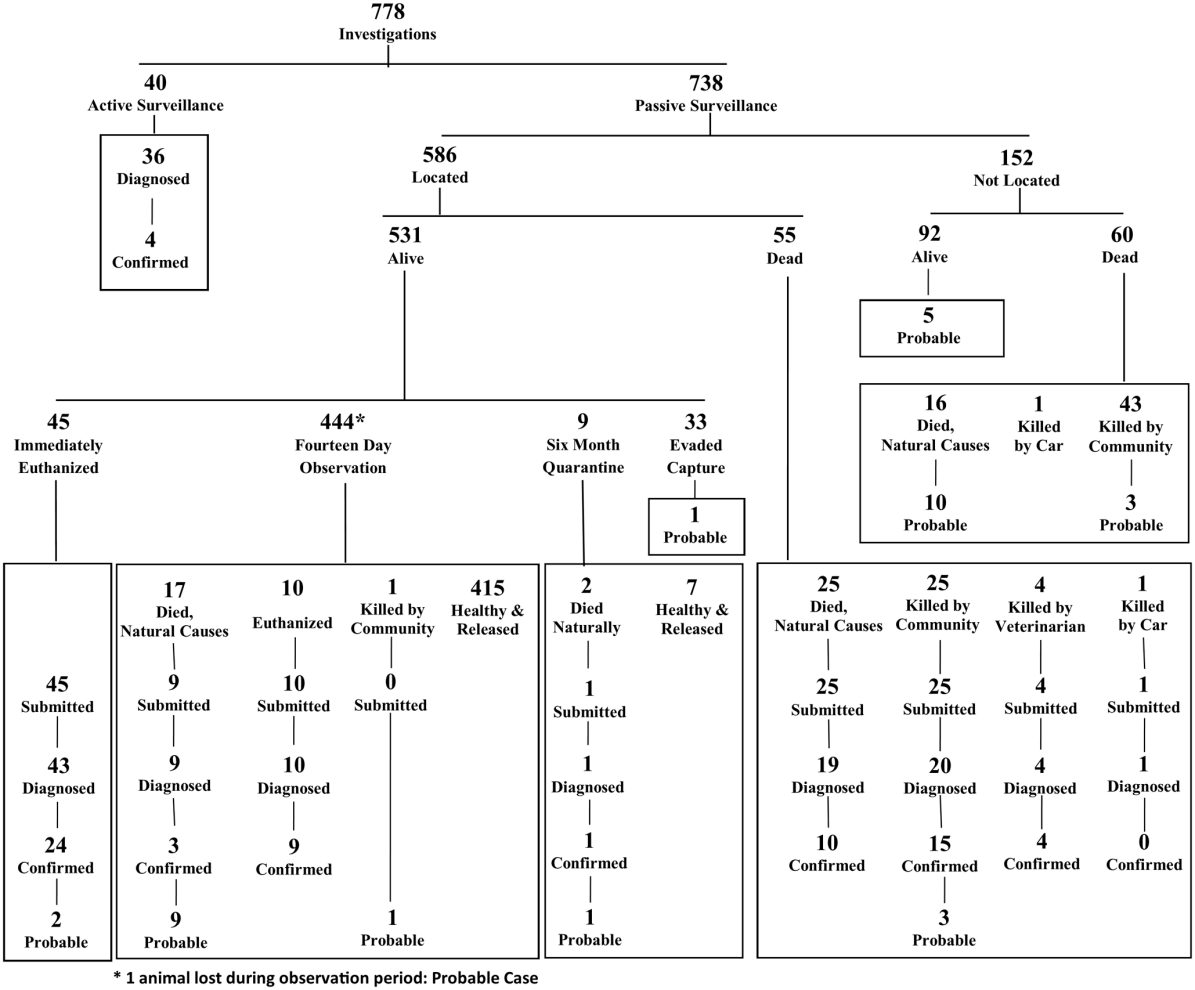
Outcomes of investigations and diagnosis of animals investigated through the Haiti Animal Rabies Surveillance Program, Haiti January 2013 –December 2014.

During the two-year period in which HARSP operated, 70 confirmed rabid animals, 36 probable, 178 suspect, and 494 non-cases were reported (Table 1). The HARSP registered 738 passive and 40 active surveillance investigations (Fig 2). Of these 778 investigations, 143 animals (18.0%) were tested for rabies and 70 (9.0%) were confirmed positive; 66 through passive surveillance and 4 through active surveillance. Of confirmed rabid animals, 62 were dogs, 4 were cats, and 4 were goats. Probable rabid animals included 33 dogs, 2 goats, and 1 pig. Overall, passive and active surveillance streams contributed 106 confirmed and probable animals (13.6% of all animals reported to HARSP for assessment).

**Table 1:** Rabies case status by animal species, Haiti January 2013 –December 2014.

Species	Case Status				Total
	Confirmed n (%)	Probable n (%)	Suspect n (%)	Non-Case n (%)	
**Dog**	58 (8.1%)	33 (4.6%)	169 (23.7%)	452 (63.5%)	712
**Dog (Active** * **)**	4 (10.0%)	0 (0.0%)	4 (10.0%)	32 (80.0%)	40
**Cat**	4 (28.6%)	0 (0.0%)	3 (21.4%)	7 (50.0%)	14
**Goat**	4 (50.0%)	2 (25.0%)	1 (12.5%)	1 (12.5%)	8
**Pig**	0 (0.0%)	1 (100.0%)	0 (0.0%)	0 (0.0%)	1
**Other**	0 (0.0%)	0 (0.0%)	1 (33.3%)	2 (66.7%)	3
**Total (N)**	**70 (9.0%)**	**36 (4.6%)**	**178 (22.9%)**	**494 (63.5%)**	**778**

* Samples collected during active, roadside surveillance activities

**Fig 3:**
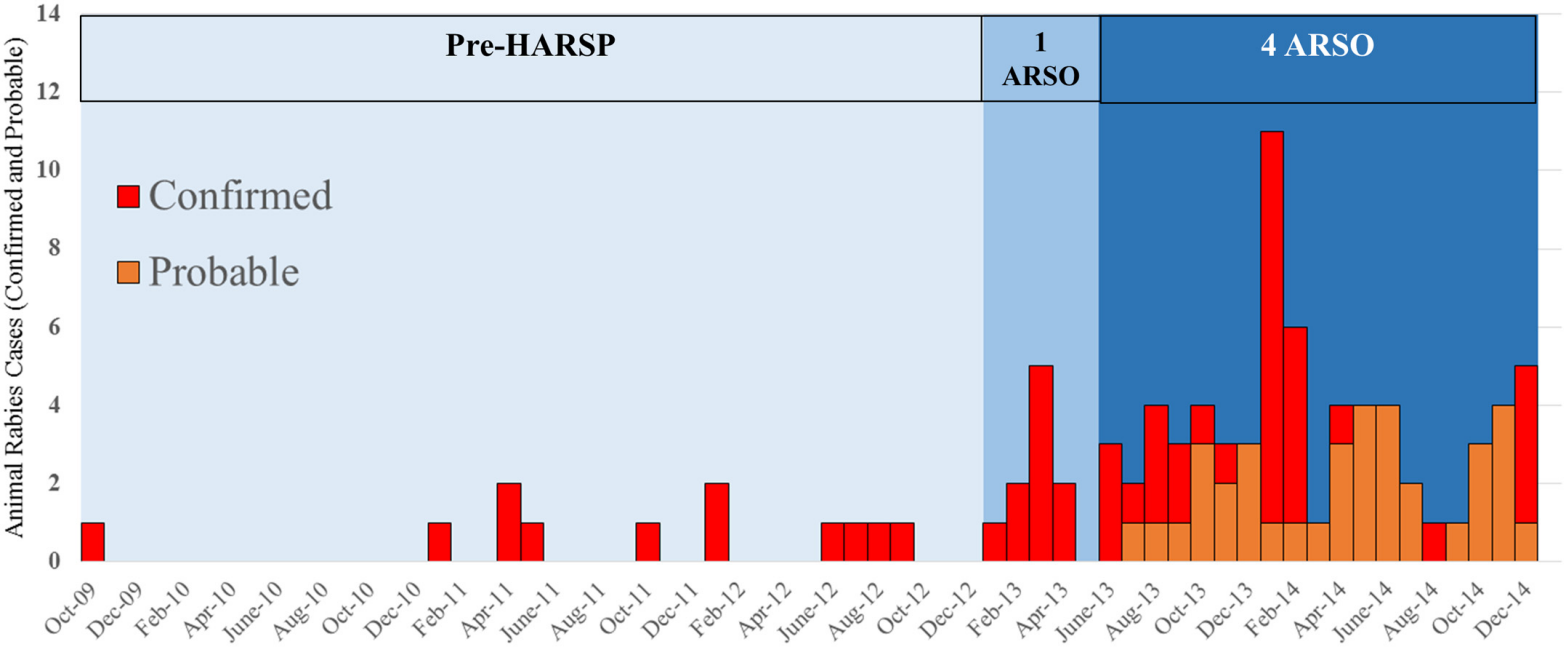
Confirmed and probable animal rabies cases by month of diagnosis, Haiti October 2009 –December 2014.

Prior to the development of HARSP, from September 2009 through December 2012, there were a total of 12 rabid animals reported in all of Haiti; an average of 0.3 rabid animals per month (Fig 3). For the first six months in which HARSP operated, 13 confirmed and probable rabies cases were reported (average of 2.2 per month), 29 during the following six months (average of 4.8 per month), and 64 during 2014 (average of 5.3 per month). The reporting rate in 2014 represented an 18-fold increase in monthly reported rabid animals compared to pre-HARSP surveillance capacity.

**Fig 4:**
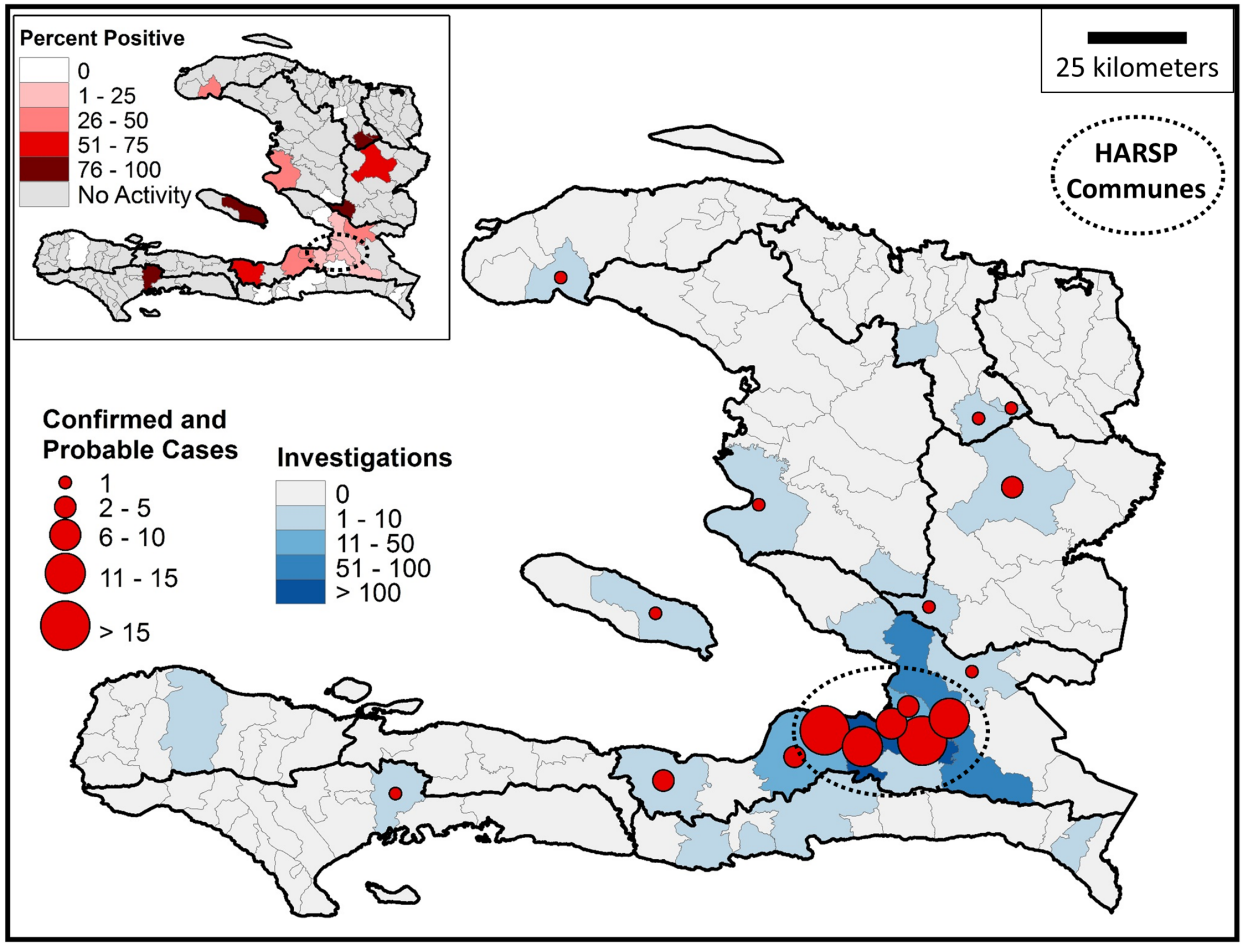
Animal rabies activity in Haiti by commune, January 2013 –December 2014.

HARSP investigations were conducted in 28 (20%) of Haiti’s 140 communes, of which rabid animals were found in 22 (78.6%) (Fig 4). The six communes without detection of a rabid animal had fewer than six investigations conducted during the two-year period. The three primary HARSP communes accounted for 507 investigations (68.5%), and resulted in the recognition of 56 (52.8%) of the 106 confirmed and probable cases. Confirmed and probable cases made up 14.5% of investigations in Petionville, 13.9% in Croix-des-Bouquets, and 6.0% in Carrefour. An average of 1.7 investigations were conducted in communes where no rabid animal had been identified, compared to an average of 36.2 investigations in communes with rabid animals. Among all communes, a confirmed or probable rabid animal was identified for every 7.4 investigations conducted.

Signs of rabies illness were recorded as part of the HARSP investigation. The most common signs of illness among confirmed and probable animal cases were unusual aggression and biting, however these signs were also common among suspect and non-cases (Table 2). Confirmed and probable cases were significantly more likely to show signs of hypersalivation (OR = 50.3 and 9.6), paralysis (OR = 6.7 and 3.6), and lethargy (OR = 9.0 and 7.3) compared to non-cases. There were no significant differences in signs of illness among suspect animals compared to non-cases. Twenty-four confirmed and probable rabid dogs died or were euthanized during the observation period, of which the average death occurred 3.0 days after observation was initiated (range 0–9 days).

**Table 2:** Clinical signs observed during investigation of rabies suspect animals by case status, Haiti January 2013 –December 2014.

Clinical Signs	Case Status											
	Confirmed			Probable			Suspect			Non-Case		
	n	(%)	OR	n	(%)	OR	n	(%)	OR	n	(%)	OR
**Aggression**	51	(72.9%)	0.8	28	(77.8%)	1.2	145	(81.5%)	1.3	378	(76.5%)	ref
**Biting**	43	(61.4%)	**0.3** **	29	(80.6%)	0.8	148	(83.2%)	0.8	419	(84.8%)	ref
**Hypersalivation**	36	(51.4%)	**50.3** **	6	(16.7%)	**9.6** **	4	(2.3%)	1.1	10	(2.0%)	ref
**Paralysis**	10	(14.3%)	**6.7** **	3	(8.3%)	**3.6** *	1	(0.6%)	0.2	12	(2.4%)	ref
**Lethargy**	7	(10.0%)	**9.0** **	3	(8.3%)	**7.3** **	2	(1.1%)	0.9	6	(1.2%)	ref
**Total (N)**	**70**			**36**			**178**			**494**		

* indicates significance at 0.05

** indicates significance at 0.01

A total of 639 bite victims were reported to HARSP and an additional 364 bite victims were identified during the course of investigations (Table 3). Of the total bite victims (n = 1,003), 137 (13.7%) were likely exposed to rabies (i.e. from both confirmed and probable cases) and 48 (35%) of these bite victims were identified as a result of the HARSP investigation. Only 43 (31.4%) people with likely rabies exposures had initiated rabies PEP at the time of the HARSP investigation; the remaining 94 persons identified as exposed were referred by the ARSOs to medical centers for further evaluation by a medical provider. While not specifically collected by HARSP, no known human rabies cases emerged from persons identified through HARSP. Two human rabies cases were detected during HARSP community-based investigation activities. In both instances the human case had died several weeks preceding HARSP activities in the community and neither cases were reported to health officials prior to HARSP detection.

**Table 3:** Human exposures to rabies suspect animals by case status and species, Haiti January 2013 –December 2014.

Animal	Case Definition	Bite Exposures				Other Exposures**				Total Exposures			
		People Bitten		People Received PEP		People Exposed		People Received PEP		Total People Exposed		Total Received PEP	
		n	%	n	%*	n	%	n	%*	n	%	n	%*
**Dog**	Confirmed	53	7.1%	6	11.3%	4	1.7%	0	0%	57	5.8%	6	10.5%
	Probable	56	7.5%	25	44.6%	18	7.7%	12	66.7%	74	7.5%	37	50.0%
	Suspect	163	21.8%	58	35.6%	39	16.7%	24	61.5%	202	20.6%	82	40.6%
	Non-Case	477	63.7%	218	45.7%	172	73.8%	52	30.2%	649	66.1%	270	41.6%
	*Total*	*749*	*100.0%*	*307*		*233*	*100.0%*	*88*		*982*	*100.0%*	*395*	
**Other**	Confirmed	5	27.8%	0	0.0%	0	0.0%	0	-	5	23.8%	0	0.0%
	Probable	1	5.6%	0	0.0%	0	0.0%	0	-	1	4.8%	0	0.0%
	Suspect	4	22.2%	4	100.0%	2	66.7%	2	100.0%	6	28.6%	6	100.0%
	Non-Case	8	44.4%	5	62.5%	1	33.3%	1	100.0%	9	42.9%	6	66.7%
	*Total*	*18*	*100.0%*	*9*		*3*	*100.0%*	*3*		*21*	*100.0%*	*12*	
**Total**	Confirmed	58	7.6%	6	10.3%	4	1.7%	0	0.0%	62	6.2%	6	9.7%
	Probable	57	7.4%	25	43.9%	18	7.6%	12	66.7%	75	7.5%	37	49.3%
	Suspect	167	21.8%	62	37.1%	41	17.4%	26	63.4%	208	20.7%	88	42.3%
	Non-Case	485	63.2%	223	46.0%	173	73.3%	53	30.6%	658	65.6%	276	41.9%
	*Total*	*767*	*100.0%*	*316*		*236*	*100.0%*	*91*		*1,003*	*100.0%*	*407*	

*row percentage, indicating the percent of people bitten, scratched, or otherwise exposed to an animal that received PEP prior to the rabies investigation. Completion of full PEP course was not ascertained.

** ‘other exposures’ include scratches and licks from rabies suspect animals.

## Discussion

### Establishment of Routine Surveillance

The rabies surveillance system developed in Haiti is based on standard surveillance practices applied to many human and animal diseases: case identification, contact tracing, epidemiologic investigation, and laboratory confirmation [14]. These are also the main principles put forth in the canine rabies blueprint, which supports that reliable and routine animal rabies diagnostic and surveillance capacity is the foundation upon which successful rabies control programs are established [10]. While the canine blueprint recommendations are readily available, it may be difficult for low-resource countries to implement all of these broad recommendations within the infrastructural confines of a developing economy. The implementation of HARSP was unique in that it was developed to operate within the infrastructural confines of both the ministries of health and agriculture, utilizing existing systems that were enhanced through the provision of targeted trainings and appropriate equipment.

The HARSP concept was originally created in 2011 as a platform in which the following targets were attempted simultaneously: diagnostics, animal surveillance, human surveillance, education, canine vaccination, and population management. These ambitious goals have been successful in developed countries where resources for such activities are readily available [6]. However, in what may be consistent with many developing countries, this multi-pronged, simultaneous approach to rabies control proved too resource-intensive for the existing infrastructure in Haiti both in terms of human and monetary capacity. In late 2012 the HARSP concept was re-focused on two main targets: diagnostic development, followed by establishment of a routine animal rabies surveillance system in three pilot communes. More resource intensive objectives, which often rely on baseline epidemiologic and surveillance acquired knowledge, were delayed until these foundational programs were established.

Establishing reliable case definitions is an integral component of any surveillance program. Animals that test positive or negative and animals that pass an observation period fall into standard case definitions: confirmed and non-case. However, animals that are not located, and those lacking diagnostic confirmation are more difficult to classify within standard case definitions. Among the four HARSP case definitions (confirmed, probable, suspect, and non-cases) there was no interpretable difference among the clinical signs of biting and aggression. This was expected, as these signs were the impetus for most HARSP investigations. Clinical illness consisting of hypersalivation, paralysis, and lethargy occurred significantly more in confirmed and probable animals than in non-cases. It is unlikely that all probable cases had rabies, but these strong associations may indicate that many animals within the probable case definition category had the disease.

Clinical signs reported among suspect rabid animals were very similar to signs reported in non-cases; perhaps an indication that rabies was unlikely within the suspect case definition group. However, suspect animals are those which were not located or assessed by ARSOs. Therefore, the clinical descriptions were based upon bite victim or community member reports, and not through the assessment of trained professionals. It is possible that a proportion of these suspect animals would have fallen into confirmed or probable case definitions had they been located and properly assessed. Overall, it would appear that the case definitions chosen for this rabies surveillance program are appropriate and should be considered for other similar surveillance programs which are trying to further the understanding of the epizootiology of endemic canine rabies.

### Animal Rabies Burden

The HARSP detected 106 confirmed and probable rabies cases in the first two years while primarily focusing efforts in just three of Haiti’s 140 communes. This two-year count represented a 10-fold increase in detection of rabid animals compared to the previous two year’s reports for the entire country, further supporting the utility of this type of surveillance model. The program appeared to improve detection capacity as time progressed, with a 140% increase in monthly case detection during the last year of the program compared to when it began. This is likely a reflection on the continued training and proficiency of ARSOs and increased community and medical facility awareness of the program. With such low rabies detection levels prior to implementation of the HARSP, and numerous competing health problems, rabies was not among the countries top health priorities. Yet, published estimation methods have predicted that there are likely 130 or more human deaths, indicating a substantial underreporting of rabies in the country [2]. The information collected through HARSP provides the first systematically collected data indicating that the animal (and likely human) rabies burden is much higher than currently recognized.

The finding that 9% of dogs assessed through passive means and 10% of dogs assessed through active means were confirmed rabid is particularly concerning. Few canine endemic countries have published the outcomes of post-bite animal rabies surveillance investigations, however several studies have published results of the rabies test percent positive during similar activities. In Haiti, rabies virus was confirmed in 66% of suspect animals which were tested. Highly endemic canine rabies affected countries have reported rates similar to or lower than what has been observed in Haiti. A study in Kenya reported 51% positive test rate among suspect cases tested, 68% in Tanzania, and 71.7% in Bhutan [3, 15, 16]. A roadside surveillance program in Kenya found 15% of found-dead dogs were positive for rabies virus, a figure comparable with what has was recorded through the HARSP [3]. These countries, Kenya, Tanzania, and Bhutan, have reported human rabies death rates ranging from 4.7–19.2 per 100,000 population [15, 16]. This study has confirmed a level of enzootic canine rabies activity in Haiti similar to that found in countries with high human rabies death rates; a finding that warrants further examination of the potential human rabies burden. If Haiti is afflicted with human rabies death rates in these comparable canine endemic countries, several hundred undocumented deaths may occur each year.

Despite HARSP being theatrically located in three communes, its actions extended to neighboring communes, where rabid animals were found in every commune where at least seven investigations were conducted. Only an estimated 8.5% of Haitians reside in the three primary HARSP communes. These communes were not chosen because of preconceived concerns about elevated rabies activity; rather they were chosen based on proximity to the national laboratory and existing infrastructure such as accessible roads and hospitals. The three communes represented both high and low socioeconomic areas, yet the highest case rate was from the wealthiest commune, Petionville (14.5%). The three HARSP communes also may have higher rates of canine rabies vaccination coverage compared to many parts of the country, as vaccination campaigns have been conducted in these communities during three of the last five years (verbal communication, MARNDR). Lastly, these communes are urban, whereas numerous studies have shown that canine rabies vaccination rates are lower and access to PEP is more difficult in rural settings [17]. Routine rabies surveillance has never been conducted in a majority of Haitian communes, including several large urban centers. Further expansion of the HARSP to serve more communes will improve the epidemiologic understanding of this disease and assist in prevention efforts.

### Benefits of Routine Animal Rabies Surveillance

#### Cessation of enzootic transmission

Rabies virus is enzootic in the canine population in Haiti, as it is in many parts of the developing world. The reproductive ratio for viral transmission in its enzootic state is relatively low (Ro = 1.01), therefore relatively basic interventions can successfully halt transmission [18]. Passive surveillance programs, besides providing valuable information for targeted interventions, may also play a role in halting enzootic transmission at the community level through the timely removal of rabid animals and reduction in canine-to-canine exposures. In the first two years of the HARSP program, 36 rabid animals were euthanized or placed into observation, preventing further animal and human exposures. This has little impact on rabies control at the national level, but may have reduced transmissions chains at the community levels. These impacts would presumably be more successful if HARSP is adopted nationally.

#### Prevention of human exposures

In many developing countries, access to veterinary medications for animal sedation and euthanasia is limited. As a result, when a rabid animal is identified in a community it must be dispatched often by crude methods that can lead to additional bites and exposures to infectious materials among those tasked with the killing. The HARSP program uses veterinary medications for sedation and euthanasia. These methods are more humane for the animals, but more importantly, are safer for the people tasked with killing the animal [13]. The 33 euthanized rabid animals represent not only interventions of the enzootic transmission chain, but also the prevention of additional human exposures encountered during crude killing methods. Despite the availability of animal assessment and euthanasia provided by HARSP, still 45% of found-dead animals had been killed by community members, presumably through traditional methods, and 75% of these animals were confirmed to have rabies. However, when animals were found alive, and owners were counselled by ARSOs, only one of 29 was killed by its owner, against HARSP recommendations. This suggests that through the education and counselling of the passive surveillance HARSP model, many potential human exposures can be prevented by offering more humane methods for removal of rabies suspect animals from communities.

#### PEP decisions improved

Over 1,000 bite victims were identified and their exposures investigated during this two year period. Rabies exposures were frequent in this cohort of bite victims, with 14% of bites being attributed to rabid animals. Healthcare seeking behaviors after an exposure to an animal suspected of having rabies was concerning. Rabies is preventable through vaccination if given in a timely manner, however nearly one-third of bite victims were only identified through active community investigations and had to be counseled on the risks of rabies and referred to medical facilities. Furthermore, of the people identified with confirmed rabies exposures, only 10% had initiated PEP at the time of the investigation (Table 3). While the HARSP does not record the motive for a person to initiate PEP, it is presumable that the implementation of active community investigation, coupled with a professional animal rabies assessment had the effect of increased PEP adherence among those with likely rabies exposures. Programs such as HARSP prevent human rabies deaths through improved bite victim identification and referral for medical assessment.

#### Sustainable use of rabies biologics

Rabies was ruled out in 494 animals through testing and observation. Rabies control programs in which animals are assessed and tested in a timely manner have the potential to reduce the unnecessary use of rabies biologics. In these types of programs, PEP may be delayed several days until testing or observation results are available. Over 92% of investigations were initiated within one day of the report and the average delay from exposure event to test result under HARSP was 7.6 days (range 1–24 days). HARSP is currently a pilot program with limited access to the majority of communities in Haiti and infrastructural limitations that can cause delays in testing and reporting of results. Therefore, it is unreasonable to delay initiation of PEP while waiting for results of an investigation. In most cases, depending on the circumstances of the bite and outcome of a risk assessment, PEP should be immediately initiated in highly endemic rabies countries [19]. If HARSP can be enhanced and expanded it may be more useful for PEP healthcare decisions. From data gathered through this surveillance program, national adaptation could result in the saving of 60% or more of rabies biologics. Haiti has experienced at least three rabies vaccine shortages from 2010 through 2015, the longest of which lasted four months in 2015. These scenarios may be avoided in the future through national adaptation of the HARSP program and better utilization of rabies vaccine stocks.

### Limitations

Passive surveillance is dependent upon reports from medical center and community channels. When these communication channels are functional, accurate extrapolation of results may be possible. The human population of the primary surveillance area was: 890,000. Hampson et al have estimated annual bite rates of 239/100,000 in developing countries such as Haiti [2]. Applying this rate to the population of the three HARSP communes, during the two-year time period we would have expected approximately 4,254 bites to have occurred in these communities. Only 738 (17%) of the estimated total bites were reported to rabies officers. This has two potential impacts on interpretation of this data. With such low reporting of bite events, there may have been more rabies activity in the community that went unrecognized. If this is the case, then the true number of canine and human rabies cases is much higher in the primary HARSP communes than currently understood. Alternatively, the event of a rabid animal in a community may be more easily recognized and reported. Under this scenario, the large percentage of bites that are not reported may be trivial bites by healthy, known dogs, in which case these surveillance data would not merit extrapolation. Increased coverage of HARSP investigations in the three primary communes could help to further the understanding of true rabies burden.

### Conclusion

Rabies is one of the world’s most feared diseases and is responsible for more deaths globally than any other zoonotic disease [1]. Yet, rabies is also a readily preventable disease with effective human and animal biologics, effective population management strategies, and effective surveillance programs that have been developed and proven economically efficient [4, 10, 20]. Unfortunately, rabies remains a neglected disease in many countries, in large part due to the cycle of neglect that starts with poor epidemiologic and epizootiologic information about the disease. Haiti is one of only four countries in the western hemisphere that still records human deaths due to canine rabies [6]. The HARSP has provided some of the first measured estimates of rabies in Haiti, and has shown that the burden and distribution of this disease is large and likely widespread. The HARSP has also shown that it can prevent human rabies deaths through active case investigations and community outreach. Programs such as HARSP are the foundation upon which successful rabies control strategies are built. The development of surveillance programs employing concepts of HARSP should be considered in countries where canine rabies remains endemic.
