# Correction: Establishment of a Canine Rabies Burden in Haiti through the Implementation of a Novel Surveillance Program

**DOI:** 10.1371/journal.pntd.0004354

**Published:** 2016-01-04

**Authors:** Ryan M Wallace, Hannah Reses, Richard Franka, Pierre Dilius, Natael Fenelon, Lillian Orciari, Melissa Etheart, Apollon Destine, Kelly Crowdis, Jesse D Blanton, Calvin Francisco, Fleurinord Ludder, Victor Del Rio Vilas, Joseph Haim, Max Millien

There is an omission in the title. The correct title is: Establishment of a High Canine Rabies Burden in Haiti through the Implementation of a Novel Surveillance Program. The correct citation is: Wallace RM, Reses H, Franka R, Dilius P, Fenelon N, Orciari L, et al. (2015) Establishment of a High Canine Rabies Burden in Haiti through the Implementation of a Novel Surveillance Program. PLoS Negl Trop Dis 9(11): e0004245. doi:10.1371/journal.pntd.0004245
